# Pembrolizumab Plus Chemotherapy in Metastatic Thymic Carcinoma: A Case Report

**DOI:** 10.3389/fonc.2021.814544

**Published:** 2022-01-20

**Authors:** Quentin Dominique Thomas, Clémence Basse, Marie Luporsi, Nicolas Girard

**Affiliations:** ^1^ Department of Medical Oncology, Montpellier Cancer institute (ICM), Montpellier, France; ^2^ Oncogenic Pathways in Lung Cancer, Montpellier Cancer Research Institute (IRCM), University of Montpellier (UM), Montpellier, France; ^3^ Department of Medical Oncology, Curie Montsouris Thorax Institute, Institut Curie, Paris, France; ^4^ Department of Nuclear Medicine, Institut Curie, Paris, France

**Keywords:** immunotherapy, immune checkpoint inhibitor (ICI), thoracic malignancies, thymic epithelial tumor (TET), therapeutic option, case report

## Abstract

Metastatic thymic carcinomas have a poor prognosis. Pembrolizumab, an anti-PD-1 antibody, has recently been evaluated for patients with metastatic thymic carcinomas progressing after at least one line of platinum-based chemotherapy. The antitumor activity of immunotherapy appears to be promising for these patients and pembrolizumab in monotherapy is actually a treatment option in second metastatic line. To the best of our knowledge, we report the first case of a patient treated for metastatic thymic adenocarcinoma with a combination of chemotherapy–immunotherapy. The patient is a 46-year-old man with metastatic thymic adenocarcinoma treated in third metastatic line with a combination of pembrolizumab plus platinum-based chemotherapy with a very good metabolic tumor response. He had a progression-free survival of 7.9 months and did not experience any severe side effects related to pembrolizumab. The association of immunotherapy and chemotherapy, as in non-small cell and small cell lung cancers, could be of interest for future therapeutic trials evaluating the survival of patients with metastatic thymic carcinoma.

## Introduction

Thymic carcinomas are a rare and heterogeneous subgroup of thymic tumors registered according to the 2015 World Health Organization (WHO) classification (1). For unresectable metastatic or recurrent tumors, exclusive chemotherapy is recommended. Standards of first metastatic line chemotherapy regimens are carboplatin area under curve (AUC) 6 + paclitaxel 200 mg/m^2^ (2, 3) or CAP [cisplatin (50 mg/m^2^), Adriamycin (50 mg/m^2^), cyclophosphamide (500 mg/m^2^)] both administered every 3 weeks intravenously (4). In a cohort of 54 patients treated by chemotherapy in first line for a metastatic thymoma or thymic carcinoma, the objective response rate was 37% for thymic carcinoma with a median progression-free survival (PFS) of 6.2 months (5).

Pembrolizumab is a humanized monoclonal antibody blocking the interaction of programmed cell death 1 (PD-1) with its ligand (PD-L1). It has been tested in two phase 2 trials for patients with thymic epithelial tumors (TETs) progressing after a first line of platinum-based chemotherapy. The median PFS was 6.1 months on a cohort of 33 patients (26 thymic carcinomas and 7 thymomas) and 4.2 months on a cohort of 40 thymic carcinomas (6, 7). These trials show encouraging results and pembrolizumab is actually a treatment option in monotherapy proposed for patient in second metastatic line with thymic carcinoma according to the National Comprehensive Cancer Network (NCCN) guidelines. There are actually no recommendations for the concomitant administration of platinum-based chemotherapy with PD-1 or PD-L1 immune checkpoint inhibitor.

We report to the best of our knowledge the first case of a patient treated in third metastatic line by chemotherapy combined with immunotherapy for a thymic carcinoma.

## Case Report

The patient, a 46-year-old man, was referred in December 2019 in our institute for a mucinous thymic adenocarcinoma with enteric flexion and neuroendocrine differentiation revealed by a painful metastasis of the right humerus. He had no significant medical history and never smoked. The Eastern Cooperative Oncology Group performance status was classified as 1. The clinical examination only revealed a functional impotence of the right shoulder due to pain, requiring morphine titration. He had no paraneoplastic manifestations of his TET, especially no myasthenia gravis and no other autoimmune disorder at baseline.

His disease was classified as stage IVb according to the eighth edition of the TNM classification proposed by the International Association for the Study of Lung Cancer (IASLC) and the International Thymic Malignancies Interest Group (ITMIG) (8). Immunohistochemistry analysis showed CK-20-positive expression. TTF1, CD-5, and CD-117 expressions were negative. CD-56 and synaptophysin were positive indicating neuroendocrine differentiation. All markers suggesting a tumor of the digestive tract or urothelial tract or germ cell tumors were negative. PD-L1 expression was 2% according to the tumor proportion score (TPS). The next-generation sequencing (NGS) panel performed on the tumor sample found a BRAF V600E mutation. The imaging assessment concluded to an oligometastatic disease with three bones sites involved. Endoscopic digestive explorations did not reveal any suspicious lesions which may suggest a primary to the digestive tract.

The patient received five cycles of carboplatin–paclitaxel in the first line until April 2020 associated with analgesic irradiations of metastatic bone lesions. Given a good response to chemotherapy and the oligometastatic status, the patient benefited from radiotherapy by proton therapy delivering 60 Gy to the primary tumor after induction chemotherapy. He did not experience any side effects related to these treatments.

A second line was initiated on bone progression in August 2020 with sunitinib 37.5 mg/day, showing poor efficacy with rapid metastatic progression on bone, pleura, and adrenal gland as detected by positron emission tomography (PET/CT) in November 2020.

**Figure 1:**
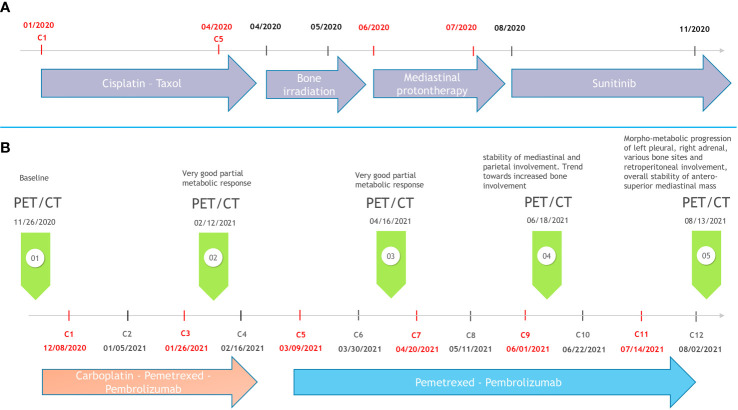
Timeline of the disease course. **(A)** Previous treatment modalities. **(B)** Third metastatic line regimen with pembrolizumab plus chemotherapy.

**Figure 2:**
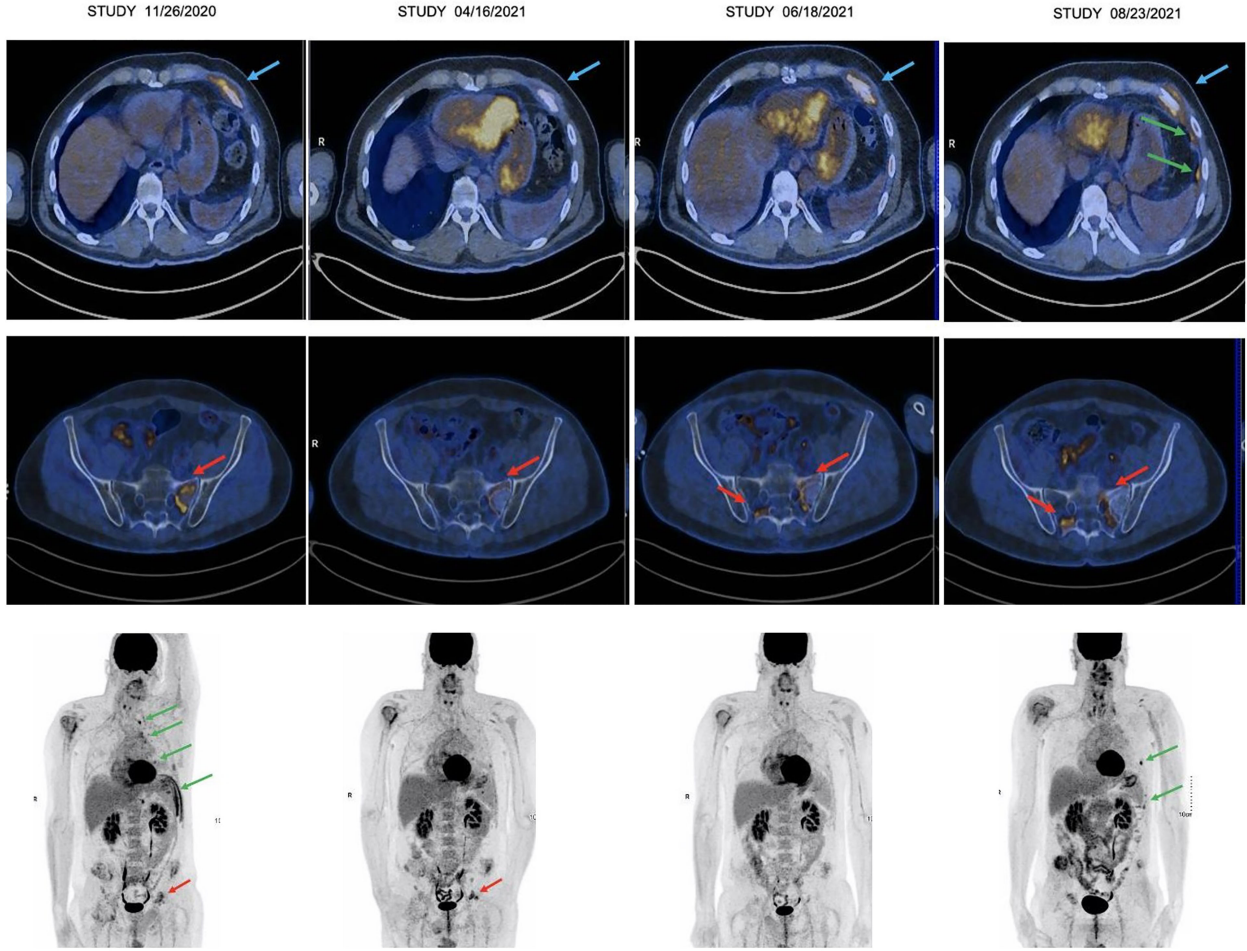
Thoracic (top) and pelvic (middle) transaxial images and whole-body PET/CT (bottom) illustrating the morphometabolic evolution. From left to right on each image: PET/CT 11/26/2020: baseline; PET/CT 04/16/2020: partial response; PET/CT 06/18/2020: maintenance of partial response with very discrete bone progression; PET/CT 08/23/2020: left pleural, soft tissues and bone morphometabolic progression. Blue arrows: soft tissue involvement; greens arrows: pleura involvement; red arrows: bone involvement.

A third line associating carboplatin AUC 6, pemetrexed 500 mg/m^2^, and pembrolizumab 200 mg was then initiated intravenously every 3 weeks. After two cycles, we observed a significant reduction of pain related to bone involvement allowing morphine withdrawal. This clinical benefit was associated to a very good partial metabolic response on PET/CT from the second cycle of chemo-immunotherapy onwards. He was treated with four induction cycles of chemotherapy–immunotherapy until February 2021. Afterwards, he received a double maintenance treatment with pembrolizumab and pemetrexed every 3 weeks, with a total of 8 cycles administered every 3 weeks until August 2021 (**Figure 1**). Unfortunately, the PET/CT performed at this time showed a dissociated response with a multimetastatic progression (on the left pleura, right adrenal gland, retroperitoneum, and various bone sites) despite the stability of the antero-superior mediastinal mass (**Figure 2**). The PFS under chemo-immunotherapy was 7.9 months. The maintenance treatment was therefore discontinued and the patient was switched to a fourth line with lenvatinib.

## Discussion

Our patient presented a PFS of 7.9 months in the third metastatic line for a thymic carcinoma treated by a combination of chemo-immunotherapy by carboplatin AUC 6, pemetrexed 500 mg/m^2^, and pembrolizumab 200 mg intravenously every 3 weeks followed by double maintenance with pemetrexed–pembrolizumab. There is currently no recommendation to propose a combination of immunotherapy and chemotherapy for metastatic TETs. Our decision was based on the aggressiveness of the disease, the fact that this combination is a standard of care for metastatic lung adenocarcinoma, and the preexisting data on the individual efficacy of chemotherapy and anti-PD-1 therapy for thymic carcinomas. This decision was also based on manageable safety profile of this combination based on trials evaluating metastatic non-small cell lung cancer (NSCLC) (9). Our patient did not experiment any severe side effects related to chemotherapy or immunotherapy. This result is encouraging compared with patients treated in first metastatic line by chemotherapy with a median PFS of 6.2 months on the RYTHMIC prospective cohort (5).

Giaccone et al. studied 40 patients treated with pembrolizumab in monotherapy after at least one previous chemotherapy regimen. In this cohort, progression-free survival was longer in patients with high PD-L1 expression (>50% of tumor cells) than those with low or no expression (median 24 months, 95% CI 5.8–42.3 versus 2.9 months, 95% CI 1.7–4.1) (7). For metastatic NSCLC, the benefit of the combination of chemotherapy and immunotherapy is found in patients with PD-L1 >50% and also with lower PD-L1 expression (9, 10). These results should be evaluated for TETs in dedicated trials to select the population benefiting from chemo-immunotherapy in this type of tumor. The benefit obtained by our patient with a PD-L1 of 2% seems to suggest that the benefit of chemo-immunotherapy is not limited to patients with a PD-L1 >50% for metastatic thymic carcinomas.

BRAF mutation is known as an oncogenic addiction with significant immunogenicity in patients with NSCLC (11). This is considered to be more significant for non-V600E BRAF mutations. Indeed, in the IMMUNOTARGET database, non-V600E mutations tended to be associated with better response rates and PFS than V600E mutations, likely due to its epidemiologic association with tobacco use compared with V600E patients (12). Our patient had a BRAF V600E mutation which may have been a factor influencing the good therapeutic response observed.

There are currently several published or ongoing trials evaluating the use of immunotherapy in the management of TETs. Published trials evaluate immunotherapy as monotherapy after at least one line of systemic treatment. Ongoing trials are evaluating doublets of immunotherapy or combinations of immunotherapy with antiangiogenic agent or chemotherapy (**Table 1**). The benefits of immunotherapy in the management of TETs seem promising, and in the coming years, it could have an important place in the management of first-line metastatic and even non-metastatic patients.

**Table 1:** Completed and ongoing trials evaluating immunotherapy in thymic epithelial tumors.

Drug regimens	Tumor type	ClinicalTrials.gov	Therapeutic line	Development stage	Patients included	Clinical outcomes
**Published clinical trials**						
Pembrolizumab IV (anti-PD-1)	Thymic carcinoma	–	After at least one previous chemotherapy regimen	Single arm phase 2	40	ORR: 22.5% (95% CI 10.8–38.5)
Pembrolizumab IV (anti-PD-1)	– Thymoma (21.2%) – Thymic carcinoma (78.2%)	–	After at least one previous platinum-based chemotherapy regimen	Single arm phase 2	33	– Global ORR: 21.2% (95% CI 10.7–37.8) – Thymoma ORR: 28.6% (95% CI 8.2–61.4) – Thymic carcinoma ORR: 19.2% (95% CI 8.5–37.9)
Nivolumab IV (anti-PD-1)	– Thymic carcinoma	–	After at least one chemo(radio)therapy	Single arm phase 2	15	ORR: 0% (95% CI 0–21.8)
Avelumab IV (anti-PD-L1)	– Thymoma (87.5%) – Thymic carcinoma (12.5%)	–	After at least one prior standard therapy (systemic therapy, thymectomy, chest radiation therapy)	Phase 1 dose escalation	8	ORR: 25%
**Ongoing clinical trials**						
Pembrolizumab IV (anti-PD-1) + Epacadostat PO (IDO1 inhibitor)	Thymic carcinoma	NCT02364076	After at least one previous chemotherapy regimen	– Single arm phase 2 – Active, not recruiting	40	ORR
Nivolumab IV (anti-PD-1) + Vorolanib PO (VEGFR/PDGFR dual kinase inhibitor)	– Thymic carcinoma – NSCLC – Refractory thoracic tumors – SCLC	NCT03583086	After any number of prior lines (no prior anti-PD-1; PD-L1 or VEGF TKI allowed)	– Phase 1/2 dose escalation and dose expansion study – Recruiting	177	– Phase 1: safety and tolerability of nivolumab and vorolanib – Phase 2: ORR/PFS/DOR
Nivolumab IV (anti-PD-1)	– Thymic carcinoma – Thymoma B3	NCT03134118	After a first platinum-based chemotherapy	– Single arm phase 2 – Recruiting	55	PFS rate at 6 months
Pembrolizumab IV (anti-PD-1) + Lenvatinib PO (multi-TKI)	– Thymic carcinoma – Thymoma B3	NCT04710628	After a first platinum-based chemotherapy	– Single arm phase 2 – Not yet recruiting	43	PFS rate at 5 months
Pembrolizumab IV (anti-PD-1) + Carboplatin–paclitaxel/nab-paclitaxel	– Thymic carcinoma – Thymoma	NCT04554524	First systemic line for locally advanced or metastatic unresectable disease	– Single arm phase 4 – Recruiting	40	ORR
Pembrolizumab IV (anti-PD-1) + Sunitinib PO (multi-TKI)	Thymic carcinoma	NCT03463460	After at least one previous regimen of platinum-based chemotherapy	– Single arm phase 2 – Recruiting	40	ORR
Pembrolizumab IV (anti-PD-1) + Cisplatin − docetaxel IV (chemotherapy)	– Thymic carcinoma – Thymoma	NCT03858582	Neoadjuvant chemo-immunotherapy for patients with unresectable thymic epithelial tumors (Masaoka stages III, IVA) followed by surgery and pembrolizumab consolidation therapy with or without radiation	– Single arm phase 2 – Recruiting	40	Percentage of major pathologic response rate defined by ≤10% of tumor composed of viable tumor
KN046 IV (bispecific PD-L1/CTLA-4 inhibitor)	– Thymic carcinoma	NCT04925947	After prior platinum-based chemotherapy and at least one immune checkpoint blockade therapy targeting PD-1, PD-L1, or CTLA-4 for locally advanced unresectable or metastatic disease	– Single arm phase 2 – Recruiting	29	Disease response rate

CTLA-4, cytotoxic T-lymphocyte antigen 4; DOR, duration of response; IDO1, indoleamine 2,3-dioxygenase 1; IV, intravenously; NSCLC, non-small cell lung cancer; ORR, partial response + complete response; PD-1, programmed death-1; PD-L1, programmed death ligand-1; PO, per os; PFS, progression-free survival; SCLC, small cell lung cancer; TKI, tyrosine kinase inhibitor; VEGF, vascular endothelial growth factor.

Particular attention should be taken for patients with metastatic TETs with frequent autoimmune myasthenia gravis associated with the diagnosis of their disease. A pre-immunotherapy autoimmune checkup should be carried out systematically, and treatment by immunotherapy should probably not be introduced in case of myasthenia gravis requiring specific treatment.

The combination of anti-PD-1 plus platinum-based chemotherapy appears to be an interesting therapeutic option which should be evaluated for metastatic thymic carcinoma in dedicated prospective trials.

## Data Availability Statement

The original contributions presented in the study are included in the article/supplementary material. Further inquiries can be directed to the corresponding author.

## Ethics Statement

Written informed consent was obtained from the individual(s) for the publication of any potentially identifiable images or data included in this article.

## Author Contributions

QT: conceptualization, writing—original draft, and visualization. CB: resources, writing—review and editing, and supervision. ML: resources, writing—review and editing, and visualization. NG: conceptualization, writing—review and editing, and supervision. All authors contributed to the article and approved the submitted version.

## Conflict of Interest

The authors declare that the research was conducted in the absence of any commercial or financial relationships that could be construed as a potential conflict of interest.

## Publisher’s Note

All claims expressed in this article are solely those of the authors and do not necessarily represent those of their affiliated organizations, or those of the publisher, the editors and the reviewers. Any product that may be evaluated in this article, or claim that may be made by its manufacturer, is not guaranteed or endorsed by the publisher.
